# Reverse Total Shoulder Arthroplasty as Treatment for Rotator Cuff-Tear Arthropathy and Shoulder Dislocations in an Elderly Male with Parkinson's Disease

**DOI:** 10.1155/2017/5051987

**Published:** 2017-08-29

**Authors:** John G. Skedros, James S. Smith, Tanner D. Langston, Micheal G. Adondakis

**Affiliations:** ^1^Department of Orthopaedic Surgery, The University of Utah, Salt Lake City, UT 84112, USA; ^2^Utah Orthopaedic Specialists, 5323 South Woodrow Street, Salt Lake City, UT 84107, USA; ^3^Intermountain Medical Center, 5121 Cottonwood Street, Salt Lake City, UT 84107, USA; ^4^University of Utah School of Medicine, 30 North 1900 East, Salt Lake City, UT 84132, USA; ^5^Tufts University School of Medicine, 145 Harrison Avenue, Boston, MA 02111, USA

## Abstract

We report the case of a 70-year-old male with Parkinson's disease (PD) and recurrent traumatic left shoulder dislocations. This case is rare because (1) he had a massive irreparable rotator cuff tear and end-stage arthritis (i.e., rotator cuff-tear arthropathy) of the same shoulder and (2) his shoulder was ultimately reconstructed with a reverse total shoulder arthroplasty (RTSA). His first dislocation occurred after a fall. Recurrent shoulder dislocations occurred despite successful closed reduction and physical therapy. Initial surgical treatment included an open capsular-labral reconstruction; RTSA was not an ideal option because of the presumed risk of failure from PD-related dyskinesias. However, the capsular-labral reconstruction failed after he lost balance and stumbled but did not fall. A RTSA was then done which restored the patient's shoulder stability and greatly improved his pain. At final follow-up two years later, he reported pain relief and improved function. This was partially attributed to the fact that he had moved to an assisted living center. He also began using an electric wheelchair one year after the RTSA. We report this case because of the unusual set of conditions and circumstances, namely, the implantation of a RTSA in a patient with PD and shoulder instability.

## 1. Introduction

Parkinson's disease (PD) is a degenerative neurological disorder that is often associated with motor symptoms (e.g., rigidity, bradykinesia, and resting tremor) that leads to postural instability, causing greater risk for falls [1, 2]. Primarily as a result of the tremors, uncontrolled body movements, and increased risk of falls caused by PD, common orthopedic procedures in these patients, including prosthetic shoulder arthroplasties, have increased complication rates with short-term results generally being more satisfactory than long-term results [3–6].

We report the case of an elderly male patient with PD and left shoulder rotator cuff-tear arthropathy with recurrent glenohumeral dislocations. Ultimately a reverse total shoulder arthroplasty (RTSA) was done to reconstruct his problematic shoulder. Our patient's case is especially unusual because patients with PD and glenohumeral arthritis often have conventional total shoulder arthroplasty (TSA) instead of RTSA. Additionally, in the United States the Food and Drug Administration (FDA) considers RTSA as an “off-label” treatment option for patients where the diagnosis extends beyond “rotator cuff-tear arthritis” (our patient had the additional diagnoses of shoulder instability and a neuromuscular disease). Our use of RTSA for our patient is consistent with the American Academy of Orthopaedic Surgeons (AAOS) belief that “surgeons may prescribe or administer any legally marketed product for an off-label use within the authorized practice of medicine in the exercise of appropriate medical judgment for the best interest of the patient” [7].

Reasons for the preferred use of conventional TSA over RTSA for some PD patients were made clear in a study of 16 TSAs in 15 patients aged 49–84 with PD in which only four patients obtained excellent results based on pain reduction, abduction, and external rotation [5]. Three patients required revision surgery due to painful subluxations and glenoid loosening. Unsatisfactory results in this study and other studies were reported primarily by patients over the age of 65 years [6, 8, 9]. Complications associated with prosthetic arthroplasties in patients with PD have been attributed to their increased muscle tone, severity of tremors, and a higher mortality rate when compared to the non-PD patients. Complications would be expected to be even higher for PD patients who have RTSA when compared to conventional TSA. This expectation is based on data showing that intraoperative, perioperative, and postoperative complications are all significantly more frequent after RTSA when compared to conventional TSA [10]. In fact, RTSA has been reported to have a complication rate four times greater than that of conventional TSA. However, it is important to note that in PD and non-PD patients most complications with RTSA are associated with revision surgery (25% after primary RTSA and 69% after revision RTSA) [11].

With these perspectives, we balanced the potential risks and benefits of using a primary RTSA to restore function and stability for our PD patient's shoulder. We report this case not only because of the good outcome but also because of the steps taken in the decision-making process that will likely help other surgeons in the management of PD patients with a similar set of circumstances.

## 2. Informed Consent

The patient was informed and agreed that data concerning the case would be submitted for publication.

## 3. Case Report

The patient is a 70-year-old right-hand-dominant male (height: 183 cm; weight: 89 kg; BMI: 26.6) who presented to our clinic in November 2014 with a chief complaint of left shoulder pain of two-year duration. It was known that he had left shoulder rotator cuff tear, left shoulder arthritis, and Parkinson's disease (PD), the latter having been diagnosed 10 years priorly. His PD was considered to be stage IV (V is worst) (stage IV = fully developed PD with severely disabling disease; the patient is still able to walk and stand unassisted but is markedly incapacitated) [14]. There was no report of significant shoulder trauma within the past 10 years and no history of dislocation. In addition to taking daily medications for PD, he was on medications for depression, hypertension, and urinary retention. These included carbidopa, ropinirole, midodrine, lamotrigine, duloxetine, nitrofurantoin, finasteride, and nortriptyline.

**Figure 1:**
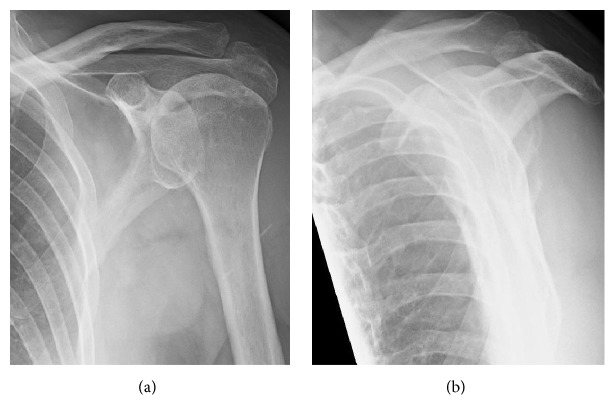
Anterior-posterior (a) and lateral (b) radiographs of our patient's shoulder prior to the arthroscopy (and prior to the dislocations).

The patient had received corticosteroid injections for pain relief in his left shoulder joint and had recently completed a physical therapy program that emphasized isometric strengthening. Physical examination showed that he had moderately reduced strength in shoulder elevation. His active range of motion (ROM) was forward flexion 105°, abduction 95°, external rotation 70°, and internal rotation 60°. The difference between active and passive shoulder elevation was 35°. Impingement and Hawkins maneuvers caused moderate pain. Radiographs of his shoulder at his initial visit to our clinic are shown in Figure 1.

Magnetic resonance scans of the patient's left shoulder showed a complete tear of the supraspinatus tendon and also of the upper one-half of the infraspinatus tendon. There were corresponding high-grade muscle atrophy, superior subluxation of the humeral head, and end-stage osteoarthritis of the glenohumeral joint. The humeral head sphericity was normal.

Because of PD-related dyskinesias, we concluded that he was not a good candidate for a reverse total shoulder arthroplasty (RTSA), which would be an ideal procedure for a non-PD patient with the same set of circumstances. In December 2015, arthroscopic debridement of his left shoulder was done by Dr. JGS. This procedure was selected because of the favorable results reported by Rockwood Jr. et al. [15] for patients with irreparable rotator cuff tears. However, this procedure provided only mild pain relief. Three months after surgery, the patient fell and dislocated the same shoulder. This was reduced in an emergency department. It was dislocated again 10 days later and could not be reduced under conscious sedation. He was taken to the operating room and Dr. JGS performed an open reduction under general anesthesia (closed reduction under general anesthesia was not possible). An open anterior capsular-labral reconstruction was then done, which included primary repair with anterior capsular shift. This included the use of three suture anchors at the glenoid rim and two at the humeral insertion.

**Figure 2:**
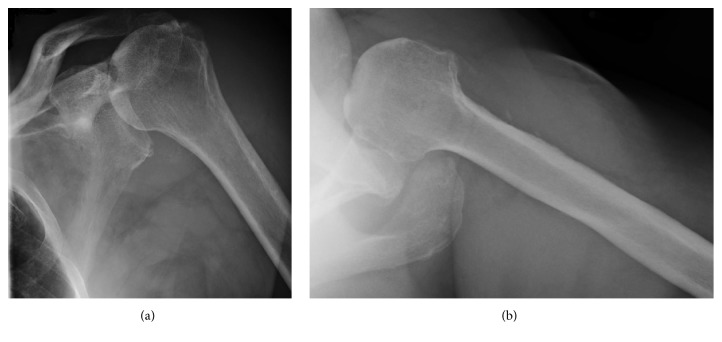
Anterior-posterior (a) and axillary lateral (b) radiographs of our patient's shoulder showing the glenohumeral dislocation.

Over the following six weeks, the patient stumbled frequently and had difficulty staying in a sling and swathe because of the PD-related dyskinesias. This caused increased pain and a sense of instability in his left shoulder. He then had another shoulder dislocation about six weeks after this soft-tissue reconstruction without having fallen (Figure 2). This caused disruption of the reconstructed soft tissues.

**Figure 3:**
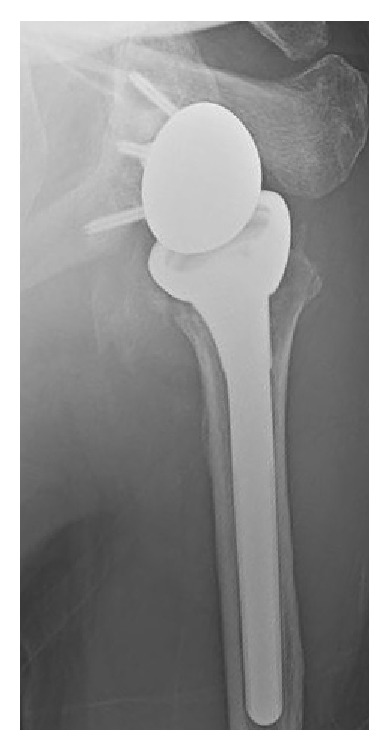
Anterior-posterior radiograph of our patient's reverse total shoulder arthroplasty at three months after implantation.

Through correspondence with John W. Sperling, M.D., an expert in shoulder arthroplasty in patients with PD [4, 16], we learned of his unpublished good-to-excellent results in PD patients who have had RTSA. Therefore in April 2015, Dr. JGS performed a noncemented RTSA on our patient using a Depuy X-TEND® (Warsaw, Indiana, USA) shoulder implant (Figure 3). Surgery was without complication and there were no significant glenoid defects and the Hill-Sachs lesion was small (Figure 2).

He recovered in an extended care facility. Despite having difficulty staying in an arm sling and falling twice, the patient had no major postoperative trauma to his left shoulder. The patient requested to continue living at the extended care facility because of increased difficulty living independently with PD. At about nine months after the RTSA the patient started showing signs of dementia, which is an additional reason for his permanent residence in the facility. Also, at 12 months after his surgery he began using an electric wheelchair because of worsening ataxia. He began transferring in and out of his wheelchair with assistance. At 18 months after the RSTA, the patient reported only mild soreness in the left shoulder with daily use.

**Figure 4:**
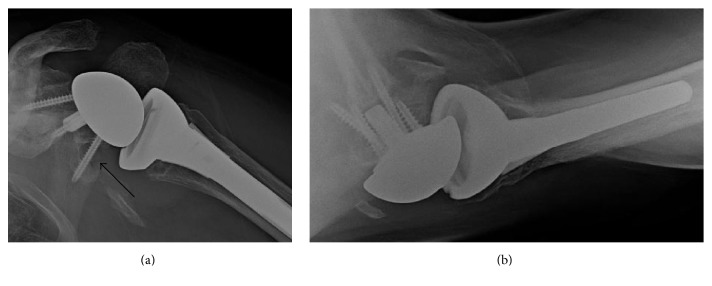
Anterior-posterior (a) and axillary lateral (b) radiographs of our patient's shoulder at two years after the reverse total shoulder arthroplasty. The arrow in image (a) indicates the glenoid notch that has formed, though no component loosing or migration has occurred. Similar findings of relatively high rates of glenoid notching are reported in PD patients [12] and in wheelchair-bound patients without PD [13].

At final follow-up at 2.5 years after the RSTA, the patient's PD had worsened; he was using an electric wheelchair for all ambulation greater than 20 meters. He was also using a walker intermittently and briefly during a typical day for short distance ambulation. He considered his replaced shoulder as “very functional”; there was no pain with activities of daily living. Active motion was 85° flexion, 80° abduction, 50° external rotation, and 45° internal rotation. Radiographs taken at his two-year follow-up are shown in Figure 4.

## 4. Discussion

RTSA is now commonly used successfully for patients who have shoulder instability with glenohumeral arthritis and an irreparable rotator cuff tear [17]. Nevertheless, one of the most common complications for patients who have RTSA is instability (Zumstein et al., 5%; Gallo et al., 15%; Edwards et al., 6%) [18–20] (none of these studies had PD patients). For patients with chronic locked shoulder dislocations, RTSA has also been used to stabilize the glenohumeral joint and restore functional use [17, 21–23]. Our patient differs from patients in these prior reports because he also had PD. Because patients with PD have asynchronous motor function and lack complete volitional muscle control, their shoulders are prone to instability events [9]. However, our patient did not describe these events during the 2.5 years since implantation of the RTSA. While this is considered a relatively short follow-up period, he has exhibited no clinical or radiographic changes to indicate that he will experience complications or treatment failure.

The usual prosthetic-replacement surgery for patients with PD who have end-stage shoulder arthritis, with or without an intact rotator cuff, is either a conventional TSA or a humeral hemiarthroplasty [4, 24]. For patients without neuromuscular diseases, RTSA is mainly used for rotator cuff-tear arthritis and for revision surgery for failed TSAs and hemiarthroplasties. RTSA is ideal for these applications because it provides a stable and fixed pivot for rotation while increasing the resting tone of the deltoid muscle, which enables it to produce active overhead arm elevation [25]. Consequently, RTSA reliably reduces shoulder pain and improves shoulder function for these patients. However in PD patients, as noted above, RTSA is much less effective in improving shoulder function than it is in relieving pain [5, 12, 26]. This is likely related to the fact that most PD patients (including ours) who have RTSA have relatively stiff shoulders [8].

Even though conventional TSA is also effective in relieving pain in PD patients with glenohumeral arthritis (without rotator cuff tear), they are more likely to fracture or otherwise reinjure their TSA-implanted shoulder when compared to non-PD patients [4, 5, 8]. For example, in a study of patients with PD from a national (USA) insurance database, Burrus et al. [8] examined 809 PD patients with shoulder arthroplasties matched with “control” non-PD patients who also had RTSA. There were no statistically significant differences between patients with PD and those without PD with respect to tobacco use, age, gender, obesity, and diabetes. Patients with PD who had RTSA had comparatively higher rates of infection, dislocation, revision shoulder arthroplasty, and systemic complications. General reasons for these high complication rates are suggested in a study of the general function of 100 patients with PD who were surveyed over the course of two months [27]. Of these 100 patients, 38 fell, 13 fell more than once a week, 13 had broken bones from these falls, and 3 were confined to a wheelchair. It has been reported that up to 90% of PD patients will sustain a fall after diagnosis [2, 27, 28]. Because of the high numbers of falls sustained by these patients a wheelchair is often recommended, as was done for our patient.

There is a relative paucity of studies in the literature regarding functional outcomes and pain improvement in patients with PD who undergo RTSA. The few studies that are available suggest that significant pain improvement can be expected with limited functional improvement. A recent matched cohort study by Cusick et al. [29] suggests that patients with PD undergoing RTSA have similar reductions in pain but inferior functional outcomes when compared to patients without PD. However, many of their results with respect to PD patients were not statistically significant due to small sample size (10 patients with PD studied). This is similar to the result seen in our patient who had complete relief of pain at final follow-up with limited improvement in shoulder function. However, their study was limited to patients with relatively mild PD (stage I or II) as opposed to the moderate-to-severe PD that was seen in our patient. They also reported a high complication rate at 40%, and fortunately our patient has not experienced complications at 2.5-year follow-up. Another case series by Dunn et al. [12] followed functional outcomes and pain relief in three patients with PD who had RTSA and reported significant pain relief measured by visual analog scale but only an average of 40° (20–60°) of forward flexion seen 17 months postoperatively. This is significantly less than the forward flexion seen in our patient at his 2.5-year postoperative follow-up (75°). Our case report contributes to this body of literature of expected outcomes with respect to pain and function in patients with PD following RTSA and raises questions that can be addressed in high quality trials with large sample sizes and long-term follow-up.

The shoulders of wheelchair-dependent patients sustain much higher load demands than normal patients. This is because manual and, often to a lesser extent, electric wheelchair-dependent patients must bear high weight when transferring into and out of the wheelchair [13]. Reduced shoulder function and/or moderate-to-severe shoulder pain as a consequence of wheelchair use can significantly reduce mobility and function of these patients. One reason for this is that when compared to normal ambulatory patients, the prevalence of rotator cuff tears is also about four times higher in wheelchair users [13, 30, 31]. In a study of 19 wheelchair-dependent patients (only one had PD) who had RTSA, Kemp et al. [13] reported that the overall complication rate is approximately 25%. Our patient's use of an electric wheelchair for nearly all ambulation activities has allowed him to avoid most early shoulder complications associated with his RTSA.

In conclusion, the published literature on RTSA in patients with PD is sparse. Our patient's case is rare because he had a RTSA in the setting of PD, rotator cuff-tear arthropathy, and recurrent glenohumeral dislocations. He had a good result that was maintained at final follow-up 2.5 years after surgery. This successful outcome was partially attributable to his use of an electric wheelchair and his permanent residence in an extended care facility.
